# Systematic symptom screening in patients with advanced cancer treated in certified oncology centers: results of the prospective multicenter German KeSBa project

**DOI:** 10.1007/s00432-023-04818-8

**Published:** 2023-05-05

**Authors:** Friederike Braulke, Servet Para, Bernd Alt-Epping, Mitra Tewes, Markus Bäumer, Birgit Haberland, Regine Mayer-Steinacker, Anne Hopprich, Maike de Wit, Michaela Grabe, Sophia Bender-Säbelkampf, Caroline Weßling, Christoph Aulmann, Christina Gerlach, Pascale Regincos, Ferdinand Fischer, Soraya Haarmann, Tatjana Huys, Sabine Drygas, Anett Rambau, Alexander Kiani, Astrid Schnabel, Christoph Buhl, Stefanie Seipke, Sonja Hiemer, Silke Polata, Maximilian Meßmann, Anna Hansmeier, Louiza Anastasiadou, Anne Letsch, Daniel Wecht, Matthias Hellberg-Naegele, Utz Krug, Ulrich Wedding, Birgitt van Oorschot

**Affiliations:** 1 Comprehensive Cancer Center, University Medical Center Göttingen, Göttingen, Germany; 2 Interdisciplinary Center Palliative Medicine, University Hospital Würzburg, Würzburg, Germany; 3 Department of Palliative Medicine, University Hospital Heidelberg, Heidelberg, Germany; 4 Department of Palliative Medicine, West German Cancer Center, University Hospital Essen, University of Duisburg-Essen, Essen, Germany; 5 Kliniken Maria Hilf GmbH Mönchengladbach, Mönchengladbach, Germany; 6 LMU Klinikum München, München, Germany; 7 Comprehensive Cancer Center Ulm. Medical Center, Ulm University, Ulm, Germany; 8 Department of Radiooncology and Radiotherapy, University Medical Center Mainz, Mainz, Germany; 9 Cancer Center Berlin-Neukölln - Vivantes Klinikum Neukölln, Berlin, Germany; 10 Cancer Center Rems-Murr-Hospital Winnenden, Winnenden, Germany; 11 Universitätsklinikum Erlangen, Erlangen, Germany; 12 Clemenshospital Münster, Münster, Germany; 13 Augsburg University Hospital, Augsburg, Germany; 14 Clinic of Hematology, Oncology and Palliative Care, Klinikum Stuttgart, Stuttgart, Germany; 15 Hospital Ludwigshafen am Rhein gGmbH, Ludwigshafen, Germany; 16 Kliniken Heilbronn GmbH, Fachklinik Löwenstein, Löwenstein, Germany; 17 Universitätsklinikum Gießen, Giessen, Germany; 18 Brüderkrankenhaus St. Josef Paderborn, Paderborn, Germany; 19 Caritas Krankenhaus Bad Mergentheim, Bad Mergentheim, Germany; 20 Klinikum Bayreuth GmbH, and Comprehensive Cancer Center Erlangen-EMN, Erlangen, Germany; 21 Leipzig University Cancer Center, Leipzig, Germany; 22 Department of Oncology, Hematology, Palliative Medicine, Special Pain Therapy, Hospital Leverkusen, Leverkusen, Germany; 23 Comprehensive Cancer Center, Hannover Medical School, Hannover, Germany; 24 Klinikum St. Georg Leipzig, Leipzig, Germany; 25 Evangelisches Waldkrankenhaus Berlin-Spandau, Berlin, Germany; 26 Department of Palliative Medicine, Hospital St. Elisabeth Straubing GmbH, Straubing, Germany; 27 Mathias-Spital Rheine-Ibbenbüren, Rheine, Germany; 28 Agaplesion Markus Krankenhaus Frankfurt, Frankfurt, Germany; 29 Department of Hematology and Oncology, University Hospital Schleswig-Holstein, Kiel, Germany; 30 Specialist Care in Oncology and Palliative Care, University Hospital Gießen and Marburg, Marburg, Germany; 31 Advanced Practice Nurse, Cantonal Hospital St. Gallen, St. Gallen, Switzerland; 32 Department of Palliative Care, University Hospital Jena, Am Klinikum 1, 07747 Jena, Germany

**Keywords:** Screening, Supportive, Palliative, MIDOS, IPOS, Cancer

## Abstract

**Purpose:**

Guidelines recommend a structured symptom screening (SC) for especially advanced cancer patients (CPs). The aim of this multicenter German prospective quality assurance project KeSBa (Kennzahl Symptom- und Belastungserfassung) was to gain knowledge on SC procedures in Oncology Centers (OCs) for advanced cancer patients and a first impression on the consequences of SC.

**Methods:**

The KeSBa project consisted of three phases: pilot, 3 months screening and feedback phase. Participating OCs decided to use either the Minimal Documentation System (MIDOS) or the Integrated Palliative care Outcome Scale (IPOS) and defined the cutoff values for positive screening results.

**Results:**

Out of 172 certified German OCs, 40 (23%) participated in the KeSBa pilot phase, 29 (16.8%) in the 3 months screening phase using MIDOS (*n* = 18, 58.6%) or IPOS (*n* = 11, 41.3%) and in the feedback round. 25/29 performed paper-based screening (86.2%). 2.963 CPs were screened. Results were documented for 1255 (42.2%, SC +) positive and 874 (29.5%, SC–) negative screenings depending on the center´s schedules: 452 SC + CPs (28.4%) and 42 SC– CPs (2.6%) had contact to specialized palliative care or other supportive specialist teams afterwards, 458 SC + CPs (28.8%) and 605 SC– CPs (38.1%) remained in standard oncology care. In the feedback round missing resources (personal and IT) and improved communication were mentioned most often.

**Conclusion:**

Routine SC is feasible in advanced CPs treated in OCs but associated with considerable workload. In 42.2% of CPs SC was classified as positive, indicating the need of further diagnostics or professional judgment. SC requires staff and IT resources.

## Introduction

In patients with cancer, especially those with advanced disease, physical symptoms and psychosocial needs are common (Vogt J et al. 2021; Culakova et al. 2023). They can be cancer-related, cancer-treatment-related or independent. National and international recommendations support a structured screening for symptoms as part of clinical routine, independent of stage and treatment goal (Hui et al. 2015; Kaasa et al. 2018; German Guideline Program in Oncology, Palliative care for patients with incurable cancer, long version 2.1, 2020). Reasons are multiple: patient tend to underreport their symptoms, a structured approach helps not to miss common symptoms, prior assessments help to focus in patient-physician-communication, deciding on the need for further assessments and involvement of other disciplines. The German Guideline for Palliative Care (German Guideline Program in Oncology, Palliative care for patients with incurable cancer, long version 2.1, 2020) recommends a systematic screening for symptoms in patients with advanced stage IV cancer. Suggested tools are the Minimal Documentation System (MIDOS) (Stiel et al. 2010) or the Integrated Palliative care Outcome Scale (IPOS) (Schildmann et al. 2016; Murtagh et al. 2019). Both, MIDOS and IPOS, are validated screening tools to assess most common physical symptoms and psychological and emotional burden. The MIDOS is based on the Edmonton Symptom Assessment System (ESAS) (Bruera et al. 1991; Stiel S et al. 2010; Hui and Bruera 2016). The integrated Palliative Care Outcome Scale (IPOS) is based on the POS (Hearn and Higginson 1999; Murtagh et al. 2019) and represents a valid, reliable and responsive tool (Murtagh et al. 2019). The ESAS is responsive and minimal clinically meaningful differences are defined, making the ESAS a suitable tool for screening and for patient-reported outcomes (PRO) (Hui and Bruera 2016). Both tools are used commonly in palliative cancer care settings for patient-reported symptom screening to decide on further assessment.

**Fig. 1:**
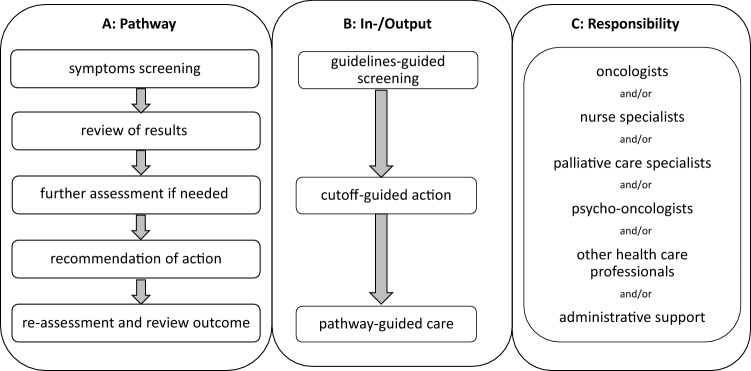
Algorithm of routine symptom assessment, endorsed by clinicians and coupled with action plans to improve clinical outcomes (adapted from Hui and Bruera 2017)

Usually, screening tools do not provide a diagnosis, but identify patients in need for more detailed assessment and diagnostic procedures or judgements of results by professionals with specialized expertise, prior to appropriate recommendations (see Fig. 1). The SCREBEL trial (Screening versus multidimensional assessment of symptoms and psychosocial distress in cancer patients from the time of incurability) (Solar et al. 2023) revealed no significant differences concerning quality of life (QoL) or survival in advanced stage cancer patients performing “low-threshold screening” using IPOS and NCCN Distress Thermometer (Mehnert et al. 2006) versus complex multi-professional symptom assessment, and the authors recommended a simple “low-threshold screening” for routine practice (Solar et al. 2023).

In centers certified as Oncology Centers according to the requirements of the German Cancer Society (Deutsche Krebsgesellschaft, DKG) and especially in certified organ-specific cancer centers for lung cancer or neuro-oncology, the use of MIDOS or IPOS to screen patients in advanced stages of the disease for symptoms is already mandatory for being certified by the DKG. In a former exploratory survey representatives of German Comprehensive Cancer Centers (CCC) were asked for structural, personal and content related barriers and facilitators of implementation of palliative care screening (Roch et al. 2021): Limitations in technical and human resources were the major barriers. In addition, a combined screening of collaborating disciplines was suggested (Roch et al. 2021).

To analyze current structures of symptoms screening in certified Oncology Centers (OCs) and to identify barriers and challenging factors for the implementation of a systematic symptom screening, the Palliative Medicine Working Group (Arbeitsgemeinschaft Palliativmedizin, APM) of the German Cancer Society (DKG, https://www.krebsgesellschaft.de/arbeitsgemeinschaften/apm.html) initiated the KeSBa project (“Kennzahl Symptom- und Belastungserfassung”, engl. Quality Indicator Symptom and Distress Evaluation) in January 2022. KeSBa was conducted together with the German Society of Palliative Care (Deutsche Gesellschaft für Palliativmedizin, DGP, https://www.dgpalliativmedizin.de/) and the working group for Oncology Nursing of the DKG (Konferenz Onkologischer Kranken- und Kinderkrankenpflege, KOK, https://www.kok-krebsgesellschaft.de/) (Para et al. 2022). The Interdisciplinary Center for Palliative Care at the University Hospital Würzburg (BvO, SP), Germany, coordinated the KeSBa project.

The aim of the multicenter prospective project was to gain knowledge on symptom screening procedures used in German certified OCs and to evaluate the feasibility of a systematic symptom screening using either MIDOS or IPOS in advanced stage cancer patients and to get a first impression about needs-oriented specialized palliative or supportive care in real-world setting.

## Materials and methods

OCs could choose symptom screening either using MIDOS (Stiel et al. 2010) or IPOS (Schildmann et al. 2015; Murtagh et al. 2019). MIDOS is a survey with questions about the intensity (no, mild, moderate, severe) of ten symptoms at the day of completion by the patient (pain, nausea, vomiting, dyspnea, constipation, fatigue, loss of appetite, tiredness, depression, fear). Other symptoms can be added, as well as a statement of general condition (Stiel et al. 2010). A sumscore of five is a cutoff value supposed in a dissertation thesis by M. M. Plöger (Plöger 2016). In addition, at least one severe symptom intensity indicates clinical relevance (van Oorschot et al. 2022). IPOS uses a questionnaire of 17 items about symptoms and concerns (pain, shortness of breath, weakness, nausea, vomiting, poor appetite, constipation, dry mouth, drowsiness, poor mobility, patient anxiety, family anxiety, depression, feeling at peace, sharing feelings, information, practical matters) measured in a 5-point numeric scale (not at all, slight, moderate, severe, overwhelming/all the time) (Murtagh et al. 2019). A higher score indicates a reduced QoL (Murtagh et al. 2019; Solar et al. 2023), and in case of at least 2 severe or 3 moderate indicated symptoms, further action is recommended (Solar et al. 2023).

The KeSBa project consisted of a pilot-phase, a three-months screening phase and a feedback round.

Pilot-phase: In January 2022 all 172 certified German OCs were asked by the German Cancer Society (DKG) to participate in the KeSBa project. Representatives (hematologists, oncologists, radio-oncologists, oncology nurses, psycho-oncologists and palliative care specialists) of 40 OCs participated in regular fortnightly online meetings. Aims of the meetings were to get information about current screening standards, to share information and experience on symptom screening with the MIDOS, IPOS or other supportive needs screening such as nutritional risk screening (NRS) or distress thermometer, and to decide on their centers participation in the upcoming three-months screening phase.

In the pilot-phase, the representatives discussed in four rounds several topics concerning documentation, responsibility for screening, standard operating procedures and cutoff values in different working groups.

As it was the aim of KeSBa to analyze structures and existing standards of symptom screening and procedures, the representatives decided for their OCs independently (a) which certified organ-specific cancer center(s) within their OCs to include in the screening-phase; (b) the use of either MIDOS or IPOS as screening tool for the three-months screening phase; (c) whether symptom screening with MIDOS or IPOS will be combined with other screening tools, e.g., distress thermometer, NRS, or others; (d) the kind of screening approach (paper-based, digitally or by interview of professionals); (e) professions involved in the completion of the symptom screening; (f) whether a training of participating professionals should happen prior the screening-phase (yes or no, if yes, what kind of training); (g) the thresholds for a positive screening within their OC or organ-specific cancer center(s); (h) professions deciding on action following a positive screening; (i) whether a query of patient´s consent prior to further assessments or interventions after positive screening(s) was necessary; and (j) the kind of consequences of a positive symptom screening (consulting of specialized palliative care professionals or other supportive care specialists or continued care by responsible oncologists or other primary oncological teams) (Table 1).

**Table 1:** Characteristics of participating centers (*n* = 29) and details on screening procedures

	CCC-OCs	Non-CCC-OCs	Total
*n* = centers	13	16	29
Screening in			
1 Organ-specific cancer center	3	7	10
2 Organ-specific cancer centers	4	3	7
3 Organ-specific cancer centers	0	0	0
4 Organ-specific cancer centers	1	0	1
> 4 Organ-specific cancer centers	3	6	9
Department of Radio-Oncology	1	0	1
Department of radio-oncology + 4 Organ-specific cancer centers	1	0	1
Screening in			
Lung cancer centers	6	5	11
Neuro-Oncology centers	5	5	10
Screening Tool			
MIDOS	7	11	18
IPOS	6	5	11
Further instruments (multiple answers possible)			
Distress and more	9	5	14
QSC-R10	0	1	1
Hornheide	0	1	1
Nutritional Risk Screening	1	1	2
No other instrument	3	6	9
Missing information	1	3	4
Approach			
Paper-based	10	10	20
Paper-based + interviews	2	3	5
Digital or APP	1	1	2
Interview by professionals	0	2	2
Performance			
Oncology nurses	2	5	7
Oncologist ± Oncology nurses	5	4	9
Oncologist + Oncology nurses + SPC	2	2	4
Oncology nurses + SPC	1	2	3
Oncologist + Oncology nurses + Psycho-oncologist	1	0	1
SPC	1	2	3
Psycho-oncologist	0	1	1
Study coordinator	1	0	1
Training	9	11	20
Responsibility for training			
Physicians	5	5	10
Nurses	0	2	2
Psycho-oncologists	0	2	2
Team approach	2	2	4
Missing information	2	0	2
Methods of training			
Learning by example	6	6	10
Handouts	2	2	4
Training seminars	2	2	4
Evaluation of screening (multiple answers possible)			
Oncology physician	7	8	15
Oncology Nurses	2	0	2
SPC physician	7	7	14
SPC nurses or other specialists	1	2	3
Psycho-oncologists	3	2	5
Study coordinator	1	0	1
Psycho-oncological support based on (multiple answers possible)			
Individual decision	2	3	5
SOP	5	1	6
Automated counseling	5	7	12
Missing information	1	4	5
SPC support based on (multiple answers possible)			
Individual decision	7	9	16
SOP	3	3	6
Automated counseling	3	4	7
Missing information	1	4	5

*CCC* comprehensive cancer center, *OC* certified oncology center, *Non-CCC-OC* certified oncology center not part of a comprehensive cancer center, *MIDOS* minimal documentation system (Stiel et al. 2010), *IPOS* integrated palliative care outcome scale (Schildmann et al. 2015; Murtagh et al. 2019), *QSC-R10* distress screening (Book et al. 2011), *APP* application, *SPC* specialized palliative care, *SOP* standard operating procedure

Screening phase: The screening phase took place from May 1th to December 31th 2022 in which the OCs collected screening data over a three-months period. According to the index of the DKG certification requirements for lung cancer and neuro-oncology cancer centers and in line with the evidence-based guideline for palliative care (German Guideline Program in Oncology, Palliative care for patients with incurable cancer, long version 2.1, 2020), all newly diagnosed stage IV cancer patients, those with relapsed or progressive disease or distantly metastasized cancer patients should receive a symptom screening when first presented in the OC. Data on screening procedures and results (participating organ-specific-cancer center; stage IV or metastasized disease according to the DKG index; screening approach (paper-based, digital-supported, interview, other); date of screening; results (positive, negative, not applicable); consequences) were collected in the OCs and were sent pseudonymously to the coordinating center in Würzburg after the end of the three-months period. Results were demonstrated according to CCC-OCs and non-CCC-OCs (Tables 1, 2, 3). Consequences of the screening were defined as assessment by professionals followed by threshold-guided interventions (e.g., SPC counseling, psycho-oncology counseling or further assessment by routine oncology care).

**Table 2:** Screening instruments and cancer entities (2963 patients)

	CCC-OCs (*n*, %)	Non-CCC-OCs (*n*, %)	Total (*n*, %)
*n* = patients (%)	1910 (64.5)	1053 (35.5)	2963 (100)
Instruments			
MIDOS	1218 (63.8)	931 (88.4)	2149 (72.5)
IPOS	692 (36.2)	122 (11.6)	814 (27.5)
Combined screening			
With psycho-oncology	724 (37.9)	301 (28.5)	1025 (34.5)
With psycho-oncology + one further screening	599 (31.6)	397 (37.7)	996 (33.6)
Certified Organ-specific cancer centers			
Lung cancer	396 (20.7)	238 (22.6)	634 (21.4)
Neuro-oncology	457 (23.9)	60 (5.7)	517 (17.4)
Colorectal cancer	108 (5.7)	140 (13.3)	248 (8.4)
Hemato-oncology	135 (7.1)	106 (10.1)	241 (8.1)
Breast cancer	49 (2.6)	98 (9.3)	147 (5.0)
Head and neck cancer	42 (2.2)	87 (8.2)	129 (4.4)
Pancreatic cancer	79 (4.1)	46 (4.4)	125 (4.2)
Skin cancer	115 (6.0)	12 (1.1)	127 (4.3)
Sarcoma	83 (4.3)	4 (0.4)	87 (2.9)
Prostate cancer	37 (1.9)	48 (4.6)	85 (2.9)
Stomach cancer	39 (2.0)	29 (2.8)	68 (2.3)
Gall bladder cancer and intestine	58 (3.0)	10 (0.9)	68 (2.3)
Endocrine malignancies	39 (2.0)	14 (1.3)	53 (1.8)
Gynecological cancer	17 (0.9)	39 (3.7)	56 (1.9)
Esophagus cancer	41 (2.1)	18 (1.7)	59 (2.0)
Urinary bladder cancer	7 (0.4)	49 (4.7)	56 (1.9)
Liver cancer	39 (2.0)	4 (0.4)	43 (1.5)
Testis and penis cancer	37 (1.9)	4 (0.4)	41 (1.4)
Kidney/renal cell carcinoma	7 (0.4)	19 (1.8)	26 (0.9)
Other	22 (1.2)	28 (2.7)	50 (1.7)
Missing information	103 (5.4)	0 (0)	103 (3.5)

*CCC* comprehensive cancer center, *OC* certified oncology center, *Non-CCC-OC* certified oncology center not part of a comprehensive cancer center, *MIDOS* minimal documentation system (Stiel et al. 2010), *IPOS* integrated palliative care outcome scale (Schildmann et al. 2015; Murtagh et al. 2019)

**Table 3:** Results of the feedback round (*n* = 29 centers, multiple answers possible)

	CCC-OCs (*n*)	Non-CCC-OCs (*n*)	Total (*n*)
Positive			
Improved communication within the KeSBa-Team	3	1	4
Improved communication within the own hospital	7	8	15
Positive feedback of patients	3	2	5
Increased awareness	3	1	4
Negative			
No distinction between treatment-related effects and tumor-related symptoms	1	0	1
No IT solutions available	5	2	7
No cutoff defined	2	1	3
No consequences defined	0	4	4
More resources necessary	8	8	16
Target group not reached	3	6	9

*CCC* comprehensive cancer center, *OC* certified oncology center, *Non-CCC-OC* certified oncology center not part of a comprehensive cancer center, *KeSBa* Kennzahl Symptom- und Belastungserfassung

Feedback round: A written survey from all participating OCs was performed asking.

- for the professions involved in screening (e.g., physicians, nurses, health care professionals, others);
- for pre-existing or new standard-operating procedures (SOPs) for screening procedures, oncology nursing counseling, need for support in implementation of a systematic screening;
- for eventually performed combined (symptom assessment, psycho-oncology, nutritional risk, others) screenings. Additionally, free text remarks were possible (barriers and facilitators). The results of the feedback survey were used to evaluate the feasibility of a systematic symptom screening.

Positive and negative free-text remarks were analyzed and clustered manually with multiple answers possible per center (FB, BvO, UW).

The central Research Ethics Commission of the University of Würzburg has confirmed that no further ethical approval is required (Nr. 20220302 01).

### Statistical analyses

Results are reported using descriptive statistics. Patients were classified as “participating patients” when at least one question of the questionnaire was answered. A questionnaire was defined as evaluable when at least 50% of questions were answered. A screening was defined as “positive” in case that screening resulted in at least one conspicuous result. In OCs with one-tool-symptom screening a “positive” screening was defined according to the individual cutoff values defined by the OCs themselves using for MIDOS or IPOS. Allocation of patients to organ-specific cancer centers and stage of the disease were defined by each OC. Data were pseudonymously stored in a central excel database, audited for accuracy, and analyzed using SPSS Version 23 for Windows (SPSS Inc., Chicago, IL) (BvO).

## Results

### Pilot phase: centers and screening characteristics

Of 172 certified OCs in Germany, 40 (23%) participated in the KeSBa pilot phase and 29 (16.8%) in the three-months screening phase and performed a symptom screening using either MIDOS (*n* = 18, 58.6%) or IPOS (*n* = 11, 41.3%). Table 1 gives an overview of centers and screening characteristics: 13/29 (44.8%) OCs were part of a comprehensive cancer center (CCC, funded as Centers of Excellence by the German Cancer Aid (Deutsche Krebshilfe, DKH)). According to the certification requirements of the DKG screening was performed in 11 lung cancer centers, 10 neuro-oncology cancer centers and further organ-specific cancer centers. Ten OCs participated with one organ-specific cancer center (34.5%), 7 further OCs (24%) participated with 2, none with 3, 1 OC (3.5%) with 4 and 9 further OCs (31%) with more than 4 organ-specific cancer centers. Additionally, one OC (3.5%) screened patients at the Department of Radio-oncology, 1 further OC (3.5%) participated with 4 organ-specific cancer center and the Department of Radio-oncology (Table 1).

For combined screening tools, 16/29 centers offered MIDOS/IPOS-screening together with psycho-social distress screening (55.1%, mostly the NCCN Distress Thermometer (Mehnert et al. 2006)), and 2/29 OCs combined three screening tools (MIDOS/IPOS screening, psycho-social distress screening and malnutrition screening, 6.8%). 25/29 OCs used a paper-based screening (86.2%) and 2/29 OCs used digital tools or APPs for patient-reported screening (6.8%). Screening was conducted mostly by the primary oncological team (physicians, oncology nurses, 25/29, 86.2%) to some extend supported by specialized palliative care (SPC) experts, psycho-oncologists or other health care professionals (e.g., social workers, study coordinators) (Table 1).

Before starting the screening phase, 20/29 OCs performed training sessions, held by physicians, nurses or by a team of two or more professions (Table 1). Training sessions included learning by example, handouts. The training sessions were done once or in up to three meetings and lasted from 15 to 30 min up to 6 h. Two OCs used quality circles for team training.

OCs could define their cutoff values of MIDOS and IPOS and the resulting consequences themselves: The IPOS-centers used the proposal of Solar et al. to identify those patients that would require further assessment (at least 2 severe or 3 moderate indicated symptoms, Solar et al. 2023). The MIDOS-centers used different sum scores (4, 5 or 6) mostly in combination with at least one or two severely pronounced symptoms or a severely reduced general condition as surrogate hint for SPC-needs.

### Screening phase: screening results and consequences

**Fig. 2:**
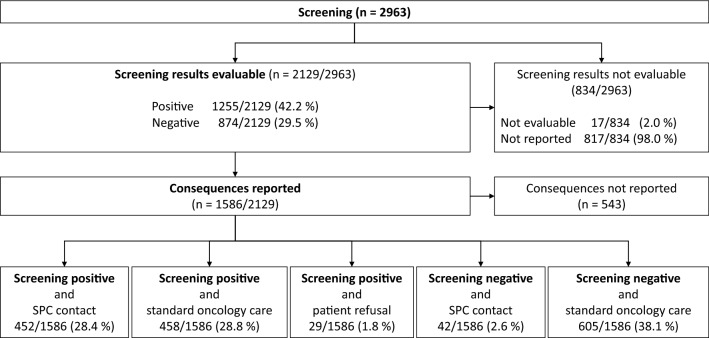
Flow Chart of the KeSBa project (*n* = 2963 patients)

During the screening phase, 29 OCs screened 2.963 adult cancer patients (mean: 102 patients/OC, median 48 patients/OC, range 7—408 patients/OC, Table 2, Fig. 2). CCC-OCs and non-CCC-OCs screened 1.910 (64.4%) and 1.053 (35.5%) of patients, respectively. Using MIDOS and IPOS, 2.149 (72.5%) and 814 (27.5%) patients were screened, respectively. In 2.021 patients a combined screening was applied together with the established psycho-social screening (68.1%, Table 2). Screening took place in 19 different organ-specific cancer centers. According to the certification requirements of the DKG, most patients were screened in lung cancer centers (*n* = 634, 21.4%) and neuro-oncology cancer centers (*n* = 517, 17.4%, Table 2). In all participating OCs the individual patient screening was evaluated for further consequences, either by members of the oncology team (10/29, 34.4%), by members of the SPC team (6/29, 20.6%) or by a psycho-oncologist (1/29, 3.4%, Table 1). In 11/29 OCs the screening was evaluated by an interdisciplinary team (37.9%) of either members of the oncological and SPC team (*n* = 5/29, 17.2%), members of the oncological and psycho-oncology team (*n* = 3/29, 10.3%) or psycho-oncology and SPC team (*n* = 3/29, 10.3%). In one OC a multi-professional team of oncological, SPC and psycho-oncology experts evaluated the screening together (1/29, 3.4%, Table 1). In total, 24/29 participating OCs (69.7%) reported screening results and consequences for patients with positive screening results: Psycho-oncological support was (in decreasing frequency) based on automated counseling based on SOPs, established SOPs and individual decision (Table 1). SPC support following a positive screening was (in decreasing frequency) based on individual decisions, automatic counseling and pre-existing written SOPs (Table 1). New SOPs for symptom screening were developed in 8 and 7 CCC-OCs and non-CCC-OCs within the KeSBa project, respectively.

**Fig. 3:**
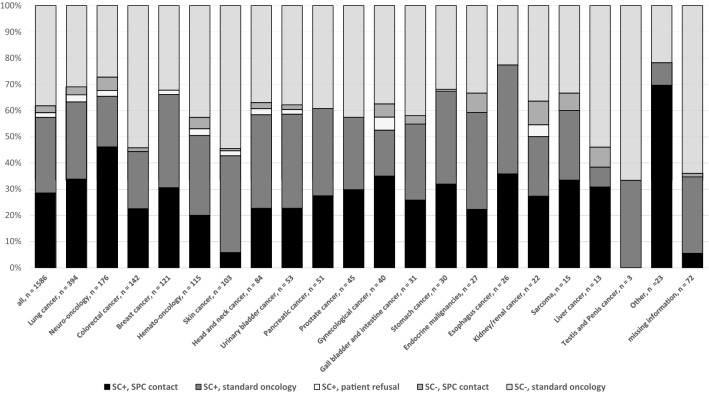
Screening results and consequences for patients with reported follow-up (*n* = 1586) according organ-specific cancer centers. *SC* + Screening positive, *SPC* standard palliative care, *SC-* Screening negative

For 834 of the 2963 screened patients, the results were not evaluable (incomplete) or not reported. Positive and negative screening results were documented for 1255 (42.2%) and 874 (29.5%) patients, respectively (Fig. 2). In the group of patients with both documented screening results and consequences (*n* = 1.586), 452 (28.4%) were counted positive and received a contact to the SPC team, 458 (28.8%) were counted positive and stayed in standard oncology care, 29 (1.8%) patients were screened positive but refused further contact, 42 (2.6%) were screened negative but received SPC contact for other reasons, and 605 patients (38.1%) were screened negative and stayed in standard oncology care (Fig. 2). Entity-specific results are shown in Fig. 3: the highest rate of positive screening was reported for patients in neuro-oncology cancer centers (46%), followed by esophageal cancer centers (36.4%), gynecological cancer (35%) and lung cancer centers (33.7%).

### Feedback remarks of participating centers

In the last feedback round centers were asked for their experiences from the pilot and screening phases and the whole KeSBa project (Table 3): on the one hand, most centers reported an improved communication within the own hospital between staff (oncology nurses, study nurses, information technology staff (IT), physicians, psycho-oncology, palliative care teams). The patients` feedback was reported to be positive and increased the awareness toward symptoms and needs of palliative circumstances, as well as an improved communication within the team. On the other hand, a lack of resources for screening was the most cited barrier, followed by problems to identify and get in touch with the target group of stage-IV cancer patients during clinical routine work. IT solutions for digital screening was missing, and consequences after positive screening were not defined. Cutoff values were rarely defined, and screening was not construed to distinguish between tumor-related symptoms and treatment-related side-effects.

## Discussion

This prospective multicenter German quality assurance project was initiated and financed by the Palliative Medicine Working Group (APM) of the DKG, in collaboration with the German Society of Palliative Care (DGP) and the working group for Oncology Nursing of the DKG (KOK) (Para et al. 2022) to evaluate the feasibility and barriers of a systematic implementation of a symptom screening in cancer patients in advanced stage according to the national guideline´s quality indicator in certified OCs. Out of 172 certified OCs in Germany, 40 (23%) participated in the pilot phase and finally 29 (16.8%) OCs screened 2.963 cancer patients in 19 different organ-specific cancer centers during the three-months screening phase. Nearly 60% of OCs chose the MIDOS screening, and more than half of the OCs combined both symptom and psycho-social screening within one screening procedure. All participating centers had to perform at least psycho-oncocolgy and malnutrition screening routinely prior to this project, otherwise they would not have been certified as OCs according to the requirements of the DKG. MIDOS or IPOS screening is routinely established in OCs within the SPC teams and units, but not for all cancer patients at advanced stage in regular oncology outpatient units—which is the new requirement of the national clinical practice guideline for palliative care (Palliative care for patients with incurable cancer, long version 2.1, 2020, AWMF-registration number: 128/001OL, https://www.leitlinienprogramm-onkologie.de/leitlinien/palliativmedizin/). As yet, the KESBA project does not provide data on the total number of advanced cancer patients treated in the OCs, but the results of structured symptom screenings will be monitored as a quality indicator within national certification programs in the following years.

Although the participating centers were mainly well-established certified OCs with various organ-specific cancer centers highly experienced in different kinds of screening programs, and 45% of them part of a CCC supported by the DKH, 86% of all centers used a paper-based questionnaire and needed additional administrative staff for screening and documentation because of lacking digital infrastructure. This was found to be a time-consuming and staff intensive process and might be a barrier for systematic routine screening (Graupner et al. 2022; Roch et al. 2021). Only two centers were able to screen via tablets or APPs in a sense of real-time patient-reported outcome measurement (PROM) (Trautmann et al. 2016; Erickson et al. 2021; Graupner et al. 2022). PROMs are helpful for enhancing patient empowerment in cancer care and can support adherence to therapy (Erickson et al. 2021). In other settings, the structured assessment of health-related quality of life (HRQoL) using PROMs enables patients to address their symptoms and needs, improved HRQoL (Velikova et al. 2004; Basch et al. 2016, 2022; Diplock et al. 2019) and even survival (Basch et al. 2022; Graupner et al. 2022). Recent studies focused on the implementation and harmonization of electronic PROMs for health care, on QoL and even on treatment-related side-effects according to the Common Terminology Criteria for Adverse Events (CTCAE) (Hagelstein et al. 2016; Hartmann et al. 2021; https://www.bundesgesundheitsministerium.de/service/publikationen/details/machbarkeitsstudie-indikationsuebergreifendes-patient-reported-outcome-measurement-digitalisierung-nutzen-fuer-eine-patientenzentrierte-gesundheitsversorgung-digiprom.html; Erickson et al. 2021; Scafa et al. 2023).

Nearly 70% of screening-active OCs performed trainings of participating physicians, nurses and health care professionals to inform about the screening phase and the upcoming procedures, which also other studies have found to be necessary (Hui et al. 2017; Hui and Bruera 2016; Kane et al. 2017; Diplock et al. 2019). OCs chose different ways to deal with the data gathered, and all individual screenings were evaluated by physicians, nurses or psycho-oncologists from oncology or specialized palliative care teams for further consequences according to pathways defined by each OC itself. Clear pathways for interventions resulting from a systematic screening and defined cutoff values are described to be essential not only in cancer patients care but also in cardiology (Hui et al. 2017; Hui and Bruera 2016; Kane et al. 2017; Kamal et al. 2020; Roch et al. 2021).

Our data show that there is an amazing acceptance and support by all participating care-givers to improve cancer care continuously, although the study centers experienced similar challenges and barriers trying to implement a systematic symptom screening in advanced stage cancer patients: according to the German guideline of palliative care (German Guideline Program in Oncology 2020) there is a quality indicator defined leading to an indicator for certified lungs cancer centers (Catalogue of requirements Lung Cancer Centers 2021) and neuro-oncology cancer center (Catalogue of requirements Neuro-oncology Cancer Centers 2020) that stage IV cancer patients should be screened for symptoms using MIDOS or IPOS. Since both MIDOS and IPOS are not yet validated to screen especially for the need of specialized palliative care but for symptoms in general, OCs within this project could choose how they perform the screening: symptom assessment alone or combined with other established screening tools for e.g., psycho-oncology, psychosocial distress or malnutrition. Our data show that in a real-life setting of in- and outpatient clinical routine it was difficult to identify the target group of stage IV cancer patients who should benefit most from a structured and systematic symptom screening. A possible option might be a combined screening of patient-reported symptoms, psycho-oncological distress screening and symptom screening using MIDOS or IPOS with symptom burden-stratified further assessment and SPC involvement according to complexity of symptoms (Kamal et al. 2020; Radbruch et al. 2020; Hodiamont et al. 2019; Grant et al. 2021).

Nearly 60% of patients showed a positive screening result, half of them got in contact with a SPC teams afterwards, half of them remained in standard oncology care without SPC support (Fig. 2). In total, 38% of patients showed inconspicuous screening results without further need of action. In contrast to a study published by Hui and colleagues (2017) with a rejection rate of 4–7% (Hui et al. 2017), only 2% of positively screened KeSBa-patients refused an intervention.

A challenging factor described by a relevant part of participating OCs within the KeSBa project was the lack of a clear cutoff value using MIDOS or IPOS. Threshold values for clinically relevant symptoms that require further action in regard of MIDOS or IPOS screening are well established and many studies evaluated cutoff values for the intensity (mild, moderate, severe) or change of symptoms in in- and outpatient settings using the ESAS score (Barbera et al. 2010; Selby et al. 2010; Stiel et al. 2010; Yennurajalingam et al. 2012; Kang et al. 2013; Hui et al. 2016; Hui and Bruera 2016), but the consequences of the screening depended on the setting. Usually those studies took place in specialized palliative care settings with SPC teams inside or outside a hospital (Stiel et al. 2010; Yennurajalingam et al. 2012; Kang et al. 2013; Zimmermann et al. 2014; Schildmann et al. 2016; Murtagh et al. 2019; Solar et al. 2023; van Oorschot et al. 2022). Zimmermann and colleagues (2014) suggested that early integration of SPC teams in addition to standard oncology care could improve quality of life and lead to minimal differences in symptoms´ intensity after 4 months (Zimmermann et al. 2014). There is less evidence in literature that ambulatory cancer patients screened positively via MIDOS or IPOS are in need of specialized palliative care as opposed to being cared for by physicians with experience in cancer care alone or by primary physicians who know the patient for a long time. In contrast to distress screening in psycho-oncology (Mehnert et al. 2006) with a clear cutoff value and a defined consequence “patient needs counseling of psycho-oncology” (German Guideline Program in Oncology 2014), this clarity for consequences of symptom screening is still topic of current research. The recently published results of the SCREBEL trial (Solar et al. 2023) showed no significant differences in QoL or survival between comprehensive assessments of QoL and symptoms versus low-threshold screening. The authors therefore preferred accessible screening tools for day-to-day clinical practice, but they also mentioned the limitation of lacking standards of consequences for positively screened patients (Solar et al. 2023). Along the lines of the Edmonton Symptom Assessment Scale (ESAS) there are different appraisals for evaluation of each MIDOS symptom or the whole questionnaire that needs further assessment (Bruera et al. 1991; Selby et al. 2010; Stiel et al. 2010; Barbera et al. 2010). The POS-Development Team proposed a detailed assessment by reported symptom impairment in the IPOS of three or four (“severe” or “overwhelming/all the time”, Murtagh et al. 2019). Hui and colleagues (2017) suggested a threshold value of ≥ 3 out of 8 items with an intensity of at least 7 for further intervention and SPC referral, but they also described a significant increase of social work counseling and psycho-oncology assessment following systematic ESAS screening (Hui et al. 2017).

The MIDOS/IPOS cutoffs for further exploration and assessment are subject of current research projects. Several proposals were discussed in the KeSBa-meetings. During the pilot phase all participating OCs used cutoffs defined by themselves in their centers. The KeSBa results show that although different cutoff values were used, the percentage of identified patients at need was comparable to former published data (Barbera et al. 2010; Kang et al. 2013; Herbert et al. 2020; Tjong et al. 2021) It was not and could not predetermined whether the centers should screen specifically for SPC needs or to identify patients with clinically relevant symptoms or burden. Cutoff values for psycho-social distress or malnutrition were taken from the established routine. Above that, using MIDOS or IPOS, the symptom assessment is not able to distinguish between unspecific tumor-related symptoms that should be addressed by SPC teams or e.g., other (primary) physicians and treatment-related side effects that need specialized oncological experience. But it helps to sensitize care-givers to identify patients at need and to monitor changes in symptom burden during follow-up by sequential assessments (Selby et al. 2010; Kang et al. 2013; Basch et al. 2016; Hui et al. 2017; Hui and Bruera 2016; Tjong et al. 2021). Our data might help to identify role models of (combined) supportive care screenings to address patient´s needs in a multi-professional team.

MIDOS or IPOS screening can serve as a tool to sensitize health care providers for symptom control, and to think about early integration of specialized palliative care in advanced stage cancer patients. On the other hand, patient-reported symptom screening might lead to more patient participation in cancer therapy.

## Limitations and strength

### Limitations

Only 29/172 certified OCs in Germany took part in the KeSBa project voluntarily, 43% of them were part of a CCCs. Results may have been confounded as engaged centers may have participated who have already implemented structured processes for supportive care screening and who have set the priority to invest time and staff in new challenges. The results of the KeSBa project are heterogeneous and summarize experiences, report individual solutions and recommendations of German certified OCs who are evidently experienced in structured supportive care screening in cancer patients. Another limitation is that no information was collected about patients who were not screened because of e.g., cognitive restrictions, reduced performance status, refusal or language barriers.

### Strengths

Thus, the results reflect the experience of experts in oncology and specialized palliative care in a real-life setting instead of a representative survey or controlled clinical trial. Feasibility in a real-world-setting was demonstrated. On the other hand, the results of the KeSBa project demonstrate that even engaged, well-structured certified OCs and CCCs suffer from similar problems with incomplete digital solutions, still paper-based and time-consuming processes and limited resources of experts in palliative cancer care. Further validation studies are needed to evaluate appropriate threshold values for further assessment and SPC involvement and other consequences of symptom screening resulting in optimal benefit for cancer patients and feasible procedures for in- and outpatient units and hospitals.

## Conclusions

Symptom screening tools such as MIDOS or IPOS may serve as a sensitization to trigger further action from care-givers and to identify patients at need. Further studies are needed to define reliable cutoff values to better identify patients at need of specialized supportive or palliative care and routine oncology or primary physician´s care. Depending on the individual way of screening different professions are involved in a screening process such as oncology nurses, study nurses, case managers, psycho-oncologists, tumor board coordinators and physicians. The KeSBa team suggests repeated teaching of all participating disciplines concerning the need of screening and the management of conspicuously screened patients following clearly defined SOPs.

## Data Availability

The datasets analyzed during the current project are available from Birgit van Oorschot, University Hospital Würzburg, Würzburg, Germany, on reasonable request.
